# *Helenus* and *Ajax*, Two Groups of Non-Autonomous LTR Retrotransposons, Represent a New Type of Small RNA Gene-Derived Mobile Elements

**DOI:** 10.3390/biology13020119

**Published:** 2024-02-13

**Authors:** Kenji K. Kojima

**Affiliations:** Genetic Information Research Institute, Cupertino, CA 95014, USA; kojima@girinst.org

**Keywords:** LTR retrotransposon, TRIM, tRNA, 5S rRNA, *Helenus*, *Ajax*, RNA polymerase III promoter, SINE, *Cassandra*, TRIMp3

## Abstract

**Simple Summary:**

Long terminal repeat (LTR) retrotransposons and non-LTR retrotransposons are two major repetitive components of eukaryotic genomes. Short intersperse elements (SINEs) are non-autonomous non-LTR retrotransposons with a sequence similar to small RNAs such as tRNA and 5S rRNA. *Cassandra* is a non-autonomous LTR retrotransposon with a 5S rRNA-like sequence in its LTRs. This observation led me to investigate the presence of non-autonomous LTR retrotransposons containing other small RNA sequences in their LTRs. Here, I discover and describe non-autonomous LTR retrotransposons *Helenus* and *Ajax*, with LTR regions of respective tRNA and 5S rRNA origin. *Helenus* and *Ajax* are likely mobilized by autonomous LTR retrotransposons. *Helenus* or *Ajax* are unlikely to encode a functional RNA. The finding of *Helenus* and *Ajax* confirms that non-autonomous LTR retrotransposons can be a source of small RNA-like interspersed sequences in the eukaryotic genomes. An understanding of their biology could contribute to the more precise prediction of small RNA genes and cellular RNA components as well as to the understanding of their evolutionary impacts.

**Abstract:**

Terminal repeat retrotransposons in miniature (TRIMs) are short non-autonomous long terminal repeat (LTR) retrotransposons found from various eukaryotes. *Cassandra* is a unique TRIM lineage which contains a 5S rRNA-derived sequence in its LTRs. Here, two new groups of TRIMs, designated *Helenus* and *Ajax,* are reported based on bioinformatics analysis and the usage of Repbase. *Helenus* is found from fungi, animals, and plants, and its LTRs contain a tRNA-like sequence. It includes two LTRs and between them, a primer-binding site (PBS) and polypurine tract (PPT) exist. Fungal and plant *Helenus* generate 5 bp target site duplications (TSDs) upon integration, while animal *Helenus* generates 4 bp TSDs. *Ajax* includes a 5S rRNA-derived sequence in its LTR and is found from two nemertean genomes. *Ajax* generates 5 bp TSDs upon integration. These results suggest that despite their unique promoters, *Helenus* and *Ajax* are TRIMs whose transposition is dependent on autonomous LTR retrotransposon. These TRIMs can originate through an insertion of SINE in an LTR of TRIM. The discovery of *Helenus* and *Ajax* suggests the presence of TRIMs with a promoter for RNA polymerase III derived from a small RNA gene, which is here collectively termed TRIMp3.

## 1. Introduction

**Figure 1:**
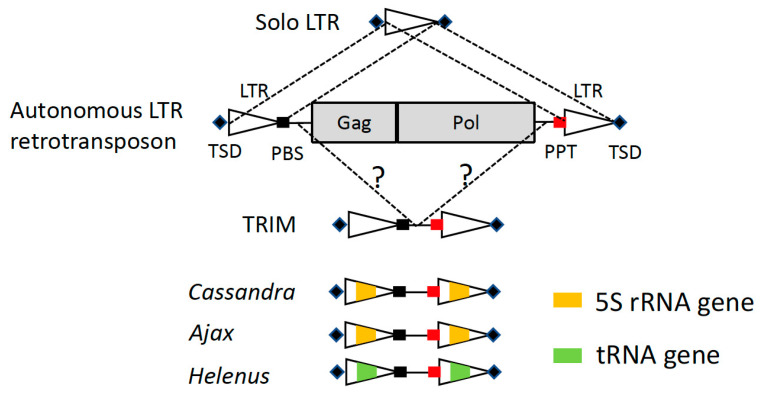
Schematic structures of autonomous LTR retrotransposons and TRIMs. LTRs are shown as triangles, while protein-coding regions are as gray rectangles. TSDs are shown as diamonds. PBSs are shown as black rectangles, and PPTs are shown as red rectangles. Small RNA-like sequences are colored in orange (5S rRNA) or in green (tRNA). Solo LTR is generated by the recombination between the two LTRs of an LTR retrotransposon. Some TRIMs are revealed to be generated by the deletion of a part of the internal portion of an autonomous LTR retrotransposon.

Retrotransposons are mobile genetic elements that propagate their copies through reverse transcription of their transcribed RNA [1,2]. Retrotransposons encode a reverse transcriptase (RT), synthesizing DNA with an RNA template. The majority of retrotransposons are classified into two groups: long terminal repeat (LTR) retrotransposons and non-LTR retrotransposons [3]. LTR retrotransposons contain two identical LTRs at both ends (Figure 1). In many eukaryotes, retrotransposons occupy a substantial portion of their genomes. The human genome contains more non-LTR retrotransposons than LTR retrotransposons [4], while most plant genomes contain much more LTR retrotransposons than non-LTR retrotransposons [5,6].

LTR retrotransposons and non-LTR retrotransposons employ two distinct mechanisms for their mobilization [7,8]. LTR retrotransposons encode an integrase and a ribonuclease H (RNase H) in addition to an RT. The transcription of an LTR retrotransposon starts inside its 5′ LTR and ends inside its 3′ LTR. It generates identical sequences (R region) at both ends of the RNA. In the mobilization of LTR retrotransposons, an RT reverse-transcribes the retrotransposon RNA using a cellular tRNA as a primer. tRNA is bound to its complementary sequence downstream of 5′ LTR, called the primer-binding site (PBS) (Figure 1). Once the DNA synthesis reaches the 5′ R region of the retrotransposon RNA, the synthesized DNA jumps to the 3′ R region, thanks to the sequence identity between two R regions, to continue reverse transcription. Once the first DNA strand is synthesized, the retrotransposon RNA is degraded by RNase H, except for the polypurine tract (PPT), which tolerates the degradation and is used as a primer for the second strand synthesis. Finally, the double-strand DNA is generated as an extrachromosomal linear DNA, and then, the DNA is integrated into the genome by the integrase. Upon integration, a short genomic sequence is duplicated at both sides of the retrotransposon (target site duplications, TSDs). Non-LTR retrotransposons do not encode an integrase but encode an endonuclease instead. In the transposition mechanism of non-LTR retrotransposons, one genomic DNA strand cleaved by an endonuclease works as a primer for reverse transcription [9]. This mechanism is called target-primed reverse transcription (TPRT). The RT of non-LTR retrotransposons is usually able to distinguish its RNA from other RNAs to initiate reverse transcription [10]. 

LTR and non-LTR retrotransposons have non-autonomous elements that do not encode an RT. Short interspersed elements (SINEs) are the dominant group dependent on non-LTR retrotransposons for their mobilization [3,11]. SINEs often contain two or more components, originating independently. The 5′ terminal portion of SINEs, called “head”, contributes to their transcription. The 3′ region, called “tail”, mimics the tail of autonomous non-LTR retrotransposons and is recognized by their transposition machinery [10]. While non-LTR retrotransposons are transcribed by RNA polymerase II, SINEs are transcribed by RNA polymerase III [12]. The majority of SINEs contain an internal promoter derived from tRNA in their heads [3]. At least two other types of promoters contribute to the transcription of SINEs. One is the 7SL RNA-derived promoter in SINE1, represented by the human *Alu* family [13]. The other is the 5S rRNA-derived promoter seen in SINE3 [14]. SINE1 and SINE3 are known to be born independently multiple times [15,16]. 

LTR retrotransposons also have short non-autonomous elements, called terminal repeat retrotransposons in miniature (TRIMs) [17] or small LTR retrotransposons (SMARTs) [18]. TRIMs were first reported from plant genomes and are widely distributed from land plants to green algae [17,19]. Outside plants, TRIMs have been reported from hymenopteran insects [20,21,22], Taeniid cestodes [23], and bivalves [24]. TRIMs are shorter than 1000 bp in length and do not encode any proteins responsible for mobilization. Still, TRIMs contain two LTRs at both sides, and in their internal region, a PBS and a PPT are found at the termini. The sequence similarity between TRIMs and autonomous LTR retrotransposons suggests that TRIMs are transposed in trans by their counterpart LTR retrotransposons that may belong to *Copia* or *Gypsy* [19]. SMARTs are the reported shortest non-autonomous LTR retrotransposon group, which are only 292 bp long and are likely mobilized by a *Copia* superfamily of LTR retrotransposon [18]. 

*Cassandra* is an extraordinary group of TRIMs; it carries a 5S rRNA sequence in its LTRs [25] (Figure 1). The sequence comparison of *Cassandra* clearly shows high sequence conservation of RNA polymerase III promoters and terminators in their 5S sequences. The transcription of *Cassandra* initiates from the 5S sequence inside of the 5′ LTR and terminates in the 5S sequence of the 3′ LTR, generating short 18 bp duplications at both ends of the RNA. *Cassandra* is distributed widely in land plants and likely dates back to the Permian, 250 MYA [26].

The sequence derived from 5S rRNA plays a role in the transcription of *Cassandra*. It is analogous to the function of the 5S rRNA sequence in SINE3. This observation led me to investigate the presence of TRIMs containing other small RNA sequences in their LTRs. Here, two new types of TRIMs, *Helenus* and *Ajax,* are reported. *Helenus* is found in fungi, animals, and plants, and includes a tRNA-derived sequence inside its LTRs. *Ajax* is found in nemertean, and its LTRs include a part of the 5S rRNA sequence. In both *Helenus* and *Ajax*, the sequence conservation is observed around the promotor motifs in small RNA-derived sequences, while their predicted secondary structures of RNA do not support their functions as either tRNA or 5S rRNA. The findings of *Helenus* and *Ajax* suggest the universality of captured small RNA sequences working as promoters in mobile genetic elements. 

## 2. Materials and Methods

### 2.1. Screening of TRIMs with tRNA-Derived Sequences

Repbase [3] was downloaded from the website of the Genetic Information Research Institute (last access: 27 November 2023). The genome assemblies were downloaded from the NCBI Assembly (https://www.ncbi.nlm.nih.gov/assembly/) (last access: 6 November 2023) (Table S1). Censor [27] searches were performed against the LTR dataset extracted from Repbase with the tRNA sequences or a subset of SINE2 sequences, both of which were also extracted from Repbase. All Censor hits were manually investigated, and only TRIMs shorter than 1000 bp in length were used for further analysis. TRIMs with tRNA-derived sequences in their LTRs were designated as *Helenus*. 

Censor searches were performed with a *Helenus* consensus sequence against the genome assemblies that are evolutionarily related to the genome harboring the respective *Helenus* family (Table S1). Censor hits were extracted and clustered with BLASTCLUST 2.2.25 in the NCBI BLAST package with the thresholds at 75% length coverage and 75% sequence identity. The consensus sequence for each cluster was generated with the 50% majority rule applied with the help of homemade scripts. Censor search was performed with the consensus sequence of each cluster against the respective genome. Up to 10 Censor hits were extracted with 500 bp flanking sequences at both sides. The consensus sequence for each cluster was regenerated to be elongated to reach both termini. The termini were determined based on the presence of LTRs and 4 to 6 bp TSDs. 

The prediction of tRNA sequences in all *Helenus* consensus sequences was performed on the tRNAscan-SE server (http://lowelab.ucsc.edu/tRNAscan-SE/index.html) (last access: 17 November 2023) [28]. All predicted tRNA sequences from *Puccinia graminis*, *Puccinia striiformis*, *Puccinia triticina*, *Uromyces viciae-fabae*, *Melampsora larici-populina*, *Cronartium ribicola*, *Saccharomyces cerevisiae*, *Aplysia californica*, *Apis mellifera*, and *Homo sapiens* were downloaded from GtRNAdb (http://gtrnadb.ucsc.edu/index.html) (last access: 21 November 2023) [29]. Sequence comparison between the predicted tRNA sequences and *Helenus* consensus sequences was performed with Censor with default parameters. 

Sequence alignment was performed with MUSCLE v.3.8.1551 [30] with default parameters. The phylogenetic tree based on the tRNA-like sequences of the fungal *Helenus* families was reconstructed using the BioNJ method implemented in SEAVIEW 4.6.1 [31], with the J-C distances and 100 bootstrapping supports.

### 2.2. Prediction of tRNA Primer Usage and Similarity

To determine PBS and PPT sequences, the 5′ and 3′ terminal 50 bp sequences of the internal portions of LTR retrotransposons from *Puccinia hordei*, *Crassostrea gigas*, *Nasonia vitripennis*, and *Danio rerio* were extracted from Repbase (last access: 27 November 2023) [3]. The 5′ terminal sequences of *Helenus* and autonomous LTR retrotransposons were aligned with MUSCLE v.3.8.1551 [30] with default parameters. The 5′ sequences were then manually compared with the tRNA datasets from GtRNAdb (http://gtrnadb.ucsc.edu/index.html) (last access: 21 November 2023) [29]. 

Sequence similarity between *Helenus* families and autonomous LTR retrotransposons was also investigated through Censor [27] searches with all *Helenus* sequences against the entire Repbase [3], which revealed the similarity between *Copia-9_PGr* and *LTR-1_PTrit*.

### 2.3. Screening of TRIMs with 5S rRNA-Derived Sequences

RepeatModeler (https://www.repeatmasker.org/RepeatModeler/) was used for the initial screening of TEs from the genome of nemertean *Notospermus geniculatus*. Censor [27] searches were performed against the genome with the consensus sequences of repeats generated by RepeatModeler. Up to 10 Censor hits were extracted with 5000 bp flanking sequences at both sides. Consensus sequences were regenerated to be elongated to reach both termini. A TRIM family with a 5S rRNA-like sequence was identified and named as *Ajax-1_NGe*. Censor searches were performed with *Ajax-1_NGe* from *N. geniculatus* against the assembly of another nemertean genome, *Lineus longissimus*. Consensus sequences were generated as described above. 

The secondary structures of LTRs of *Ajax* were predicted on the RNAfold web server (http://rna.tbi.univie.ac.at/cgi-bin/RNAWebSuite/RNAfold.cgi) (last access: 28 November 2023) and were compared with the secondary structures reported in 5SrRNAdb (http://combio.pl/rrna/) (last access: 28 November 2023). 

All reported consensus sequences for *Helenus* and *Ajax* are available in the Supplementary Materials as well as in Repbase (https://www.girinst.org/repbase/) [3]. 

### 2.4. Characterization of SINE3 from Lepidopteran Genomes

The genome assemblies of four lepidopteran insects (the common copper brome *Lycaena phlaeas* (ilLycPhla1.1), the garden grass-veneer *Chrysoteuchia culmella* (ilChrCulm1.1), the dusky thorn *Ennomos fuscantarius* (ilEnnFusc2.1), and the ringed china-mark *Parapoynx stratiotata* (ilParStra1.1)) were downloaded from the NCBI Assembly (https://www.ncbi.nlm.nih.gov/assembly/) (last access: 6 November 2023). Censor [27] searches were performed with the *HaSE3* consensus sequence against the genome assemblies. Consensus sequences were generated as described above. The termini were determined based on the sequence similarity to *HaSE3* or other SINEs in Repbase [3]. 

Sequence alignment of *Ajax*, SINE3, and 5S rRNA sequences was performed with MUSCLE v.3.8.1551 [30] with default parameters. 

### 2.5. Estimation of Copy Numbers

Censor [27] searches were performed with six genomes (*P. triticina*, *C. ribicola*, *D. rerio*, *C. gigas*, *N. vitripennins*, and *L. longissimus*) as queries against their respective *Helenus/Ajax* consensus sequences. The hits longer than 90% of the respective consensus and with over 90% sequence identity were assumed as full length. The LTR and the internal portion were counted independently. 

## 3. Results

### 3.1. Helenus: TRIM with a tRNA-Derived Sequence

Censor [27] searches with tRNA and *SINE2* sequences against LTRs in Repbase [3] revealed that not a few LTRs contain a sequence similar to a part of tRNA. Among them, *LTR-1_PGr* from the rust *P. graminis*, *LTR-1_PSt* and *LTR-2_PSt* from *P. striiformis*, *LTR-1_PTrit* from *P. triticina*, *LTR1_LCh* and *LTR2_Lch* from the coelacanth *Latimeria charmnae*, *LTR-11_DR* from the zebrafish *D. rerio*, and *ULTR-1_NVit* from the jewel wasp *N. vitripennis* could be classified as TRIMs based on their lengths and structures (Table S1). Here, the TRIMs with tRNA-derived sequences in their LTRs are designated as *Helenus*, the twin brother of *Cassandra*. 

### 3.2. Helenus from Fungi

Using characterized *Helenus* sequences as seeds, the extensive analysis to characterize LTRs with tRNA-derived sequences was performed (Table 1 and Table S1). In fungi, Pucciniomycetes including *P. graminis*, *P. striiformis*, and *P. triticina* harbor many *Helenus* families. *Helenus* families were characterized in the genera *Puccinia*, *Austropuccinia*, *Melampsora*, *Uromyces*, *Cronartium*, and *Hemileia*, all in the order Pucciniales. Outside of Pucciniales, no *Helenus* was found from Pucciniomycetes, including *Microbotryum*, *Leucosporidium*, and *Rhodotorula*. *Helenus* was also detected from several Agaricomycetes fungi: *Agaricus bisporus*, *Coprinopsis cinerea*, and *Amylostereum areolatum*. In total, 113 *Helenus* families were found from Pucciniales fungi, and multiple *Helenus* families co-exist in the same genome. Over 10 *Helenus* families were found from *P. coronata*, *P. horiana*, *P. triticina*, and *C. ribicola*. These results suggest that *Helenus* has been maintained in the genomes of Pucciniales fungi. Pucciniales fungi date back to the Jurassic, ~130 million years ago (MYA) [32]. 

Some *Helenus* families retain no full-length copies, but rather fragments and solo LTRs, such as *Helenus-6_PSt* and *Helenus-7_PHord* (Table S1). Many of the solo LTRs of *Helenus* are flanked by 5 bp TSDs as they have experienced LTR-LTR recombination after transposition. 

### 3.3. Helenus from Animals

In animals, six phyla (Arthropoda, Brachiopoda, Chordata, Ctenophora, Mollusca, and Nemertea) include at least one species whose genome maintains *Helenus* (Table 1 and Table S1). In Brachiopoda, *Lingula anatina* contains at least three families of *Helenus*. The genome of *Mnemiopsis leidyi* in Ctenophora contains a *Helenus* family. In Nemertea, two related ribbon worms *L. longissimus* and *N. geniculatus*, both in the family Lineidae, have *Helenus* families. 

In Arthropoda, using *ULTR-1_NVi* as a seed, diverse *Helenus* families were characterized from six families of parasitoid jewel wasps (Pteromalidae, Eurytomidae, Eupelmidae, Megastigmidae, Cynipidae, and Torymidae) in the superfamily Chalcidoidea, Hymenoptera. In total, 40 *Helenus* families were found from 14 genomes. No *Helenus* family was found in Trichogrammatidae, Ormyridae, Eulophidae, Aphelinidae, Mymaridae, Eucyrtidae, Agaonidae, or Chalcididae in Chalcidoidea. The common ancestor of the wasps with *Helenus* dates back to the Early Cretaceous, over 100 MYA [33]. Hymenopteran *Helenus* families include the longest family among *Helenus* found in this study. The longest *Helenus* family is *Helenus-1_SyUm* from *Synergus umbraculus*, which is 1263 bps. Its internal portion is also the longest, 621 bps, and its LTRs are the second longest, 321 bps, only two bps shorter than the LTR of *Helnus-1_SyJa*, 323 bps from *Synergus japonicus*. 

In Chordata, the genome of coelacanth *L. chalumnae* contains two families of *Helenus*: *LTR1_LCh* and *LTR2_Lch* [34]. The reported consensus sequences of these two families include both LTRs and the internal sequence. The sterlet *Acipenser ruthenus* and American paddlefish *Polyodon spathula* are distant relatives but contain closely related *Helenus* families whose consensus sequences are ~98% identical to each other. *LTR-11_DR* from the zebrafish *D. rerio* also shows similarity to tRNA sequences in its LTRs. *Helenus* families related to *LTR-11_DR* are found in various Cypriniformes genomes and the two genomes outside of Cypriniformes: Asian redtail catfish *Hemibagrus wyckioides* (Siluriformes) and the milkfish *Chanos chanos* (Gonorynchiformes). These three orders belong to the superorder Ostariophysi. Some copies of *Helenus-1_CaAu* from the goldfish *Carassius auratus* are ~99.5% identical to their consensus, showing its very recent activity. On the other hand, these *LTR-11_DR*-related *Helenus* families are very conserved in sequence. *Helenus-1_CaAu* has the internal portion completely identical to that of *LTR-11_DR*. The distribution and sequence conservation indicate that *LTR-11_DR*-related *Helenus* families date back to the Triassic, >200 MYA [35]. 

In Mollusca, bivalves in the five orders (Ostreida, Pterioida, Mytilida, Pectinida, and Arcoida) in the subclass Pteriomorphia contain at least one *Helenus* family in their genomes. In total, 51 *Helenus* families were found from these five orders. Limoida, the other order in Pteriomorphia, includes no available genome assembly as of Nov. 22, 2023. The wide distribution in Pteriomorphia suggests the origin of *Helenus* in the common ancestor of Pteriomorphia, which dates back to the Early Ordovician, over 470 MYA [36]. 

### 3.4. Helenus from Plants

The only plant species with a characterized *Helenus* family is *Selaginella moellendorffii* (Table 1 and Table S1). The LTR of *Helenus-1_SMo* is 120 bp in length, and its internal portion is only 85 bp in length. It generates 5 bp TSDs upon integration.

### 3.5. tRNA-Derived Sequences in Helenus

**Figure 2:**
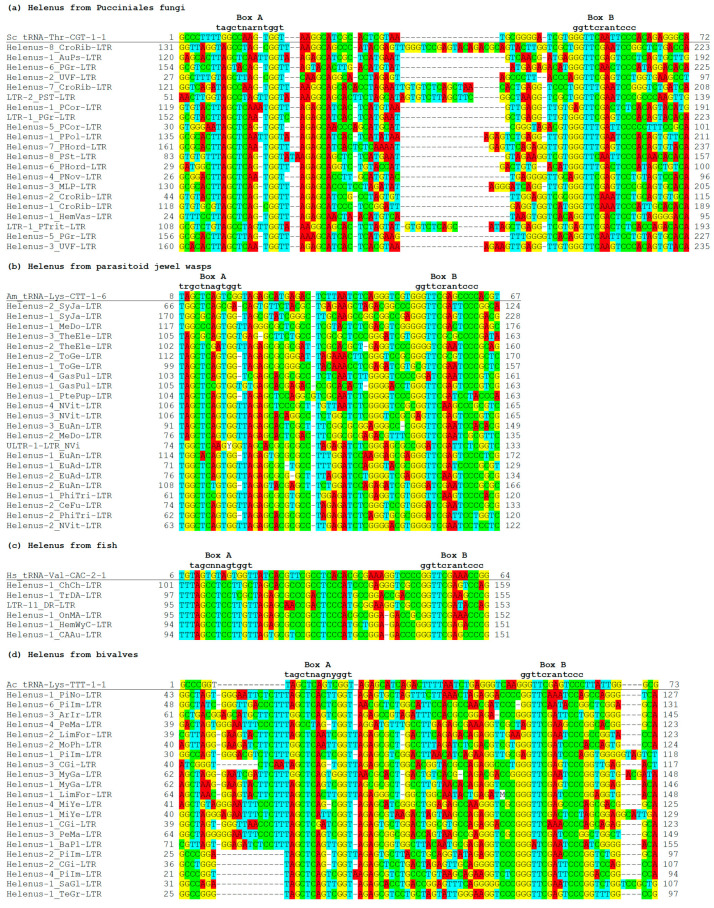
tRNA-derived sequences in *Helenus* LTRs. Boxes A and B for RNA polymerase III promoter are shown above the alignment with their motif sequences. The positions of the aligned sequence in the respective consensus is shown at both sides. (**a**) tRNA-derived sequences in representative fungal *Helenus* LTRs aligned with tRNA-Thr-CGT from *Saccharomyces cerevisiae*. (**b**) tRNA-derived sequences in representative hymenopteran *Helenus* LTRs aligned with tRNA-Lys-CTT from *Apis mellifera*. (**c**) tRNA-derived sequences in representative fish *Helenus* LTRs aligned with tRNA-Val-CAC from *Homo sapiens.* (**d**) tRNA-derived sequences in representative molluscan *Helenus* LTRs aligned with tRNA-Lys-TTT from *Aplysia californica*.

The comparison between tRNA genes and *Helenus* LTRs revealed the conservation around boxes A and B, responsible for the RNA polymerase III transcription (Figure 2). Most *Helenus* LTRs contain TRGCY and YGG in box A, and GGTTC is conserved in box B. When tRNAScan-SE [28] is used to determine the types of tRNA sequences in *Helenus* LTRs, the highest scoring isotype model does not match the anticodon-inferred isotype in many *Helenus* families. Such isotype prediction disagreement (IPD) indicates that the predicted tRNA is unfunctional. When excluding these cases, only a few *Helenus* from bivalves and fungi are predicted to encode a tRNA (Figure S1). In bivalves, seven *Helenus* families can encode a tRNA-Thr. In fungi, 15 *Helenus* families can encode a tRNA for His or Thr. This raises the possibility that *Helenus* contributes to the expansion of tRNA genes. 

GtRNADB (http://gtrnadb.ucsc.edu/) (last access: 21 November 2023) [29] reports substantial numbers of predicted tRNA genes in Pucciniales fungi. *P. triticina* is predicted to have 1538 tRNA genes, including 225 predicted pseudogenes. They include 492 tRNA-Thr-AGT, 189 tRNA-Ser-TGA, 137 tRNA-Ala-TGC, and 128 tRNA-Pro-TGG. *P. striiformis* is predicted to have 890 tRNA genes including 138 predicted pseudogenes. *P. graminis* is predicted to have 506 tRNA genes including 103 predicted pseudogenes. Outside the genus *Puccinia*, *Uromyces viciae-fabae* contains 588 predicted tRNA genes, and *Melanopsola larici-populina* has 251 predicted tRNA genes. *Cronartium ribicola* is predicted to have 1670 tRNA genes, including 519 predicted pseudogenes. No predicted tRNA dataset is available for mollusks except *Aplysia californica* in GtRNADB. 

There is an expansion of certain types of tRNA genes in Pucciniales fungi. In the genus *Puccinia*, tRNA-Thr-AGT, tRNA-Ser-TGA, tRNA-Ser-AGA, tRNA-Ala-TGC, tRNA-Ala-AGC, and tRNA-Pro-TGG are predicted in extremely high copy numbers. Many of these predicted tRNA genes correspond to the tRNA-like sequences in one or more *Helenus* families (Table 2). When excluding predicted tRNA genes in *Helenus* copies, only a small number of predicted tRNA genes remain in each *Puccinia* genome. In the genera *Uromyces* and *Cronartium*, numerous genes for tRNA-Thr-AGT, tRNA-Ser-AGA, tRNA-Sup-CTA (suppressor tRNA), tRNA-Ala-AGC, and tRNA-Val-GAC are predicted; however, after excluding predicted tRNA genes showing high similarity to *Helenus*, less than 10 genes for each tRNA type remain in each genome. In the cases of tRNA-Sup-CTA, all predicted genes originate from *Helenus*. 

Among the *Helenus* families that show sequence similarity to predicted tRNA genes of high copy number (Table 2), only two families, *Helenus-4_PSt* and *Helenus-4_PGr,* are able to encode a functional tRNA (Figure S1). They may contribute to the tRNA gene increase by 21 and 15, respectively (Table 2). Even if they are functional, their contribution to the tRNA gene increase is limited. *Helenus* is less likely to contribute to the diversity and expansion of functional tRNAs, although functional validation is needed to eliminate that possibility. 

### 3.6. Primer-Binding Sites (PBSs) of Helenus

A primer-binding site (PBS) is located near the 5′ terminus of internal regions, just downstream of the junction of the 5′ LTR. Most LTR retrotransposons and retroviruses use the 3′ end of a tRNA as a primer for reverse transcription, and the PBS is often a sequence complementary to the 3′ end of tRNA. Hymenopteran *Helenus* families use the 3′ end of either tRNA-Lys, tRNA-Leu, tRNA-Trp, or tRNA-Arg as a primer, determined based on the sequence complementarity between the PBS and tRNA (Figure S2A). Vertebrate *Helenus* families use the 3′ end of tRNA-His, tRNA-Leu, or tRNA-Lys (Figure S2B). *Helenus* families from bivalves use the 3′ end of tRNA-Lys, tRNA-His, tRNA-Ile, tRNA-Pro, tRNA-Glu, or tRNA-Ser as a primer (Figure S2C). 

**Figure 3:**
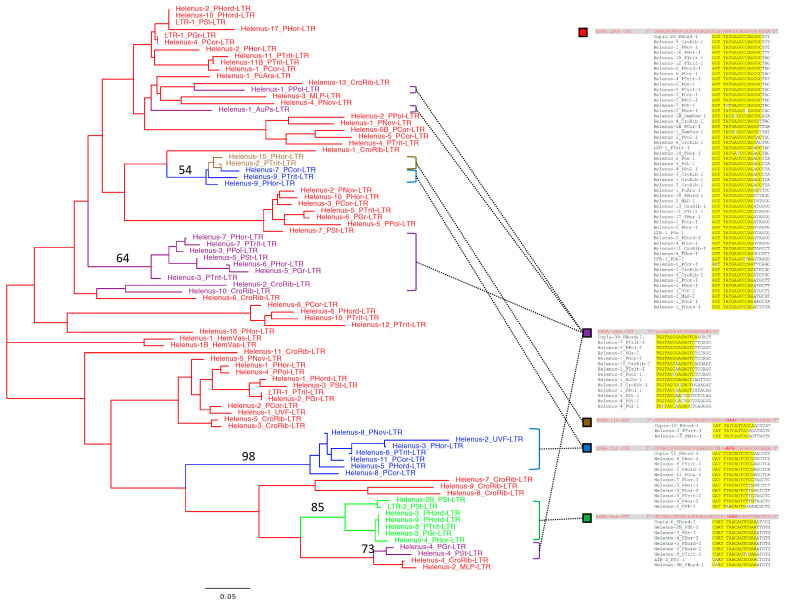
The evolution and PBS sequences of the fungal *Helenus* families. The phylogeny shown on the left is based on the tRNA-derived sequences in *Helenus* LTRs. The line and leaf color indicate the primer tRNA type. The bootstrap support value over 50% is shown at the branch of *Helenus* families not using 3′-truncated tRNA-iMet-CAT as a primer. PBS sequences are shown on the right. tRNA sequences are shown in red and in the reverse orientation. The anticodons are shown in bold. The site of intron is indicated by “|”. The PBS nucleotides complementary to the tRNA are highlighted in yellow.

In contrast, only several fungal *Helenus* families use the 3′ end of tRNA as a primer for reverse transcription. The majority of fungal *Helenus* families contain PBSs complementary to the anticodon loop of tRNA (Figure 3). The anticodon loop sequence of tRNA-Tyr, tRNA-Ile, tRNA-Asp, and tRNA-iMet appears to be used as a primer for reverse transcription. These four types of tRNA genes contain an intron downstream of their anticodons. The PBS sequences suggest the usage of mature tRNA (without intron) as a primer for reverse transcription.

*Helenus* and some autonomous LTR retrotransposons show sequence similarity at the PBS region. Only autonomous *Gypsy* LTR retrotransposons show similarity to the PBS region of animal *Helenus* families. In bivalves, *Gypsy-125_CGi* uses tRNA-Lys as a primer, *Gypsy-39_CGi* uses tRNA-His, *Gypsy-157_CGi* uses tRNA-Ile, and *Gypsy-13_CGi* uses tRNA-Pro (Figure S2C). In insects, *Gypsy-16_NVi* uses tRNA-Lys as a primer for reverse transcription, *Gypsy-28_NVi* uses tRNA-Leu, *Gypsy-32_NVi* uses tRNA-Trp, and *Gypsy-31_NVi* uses tRNA-Arg (Figure S2A). 

Unlike animal *Helenus* families, fungal *Helenus* families show sequence similarity at the PBS region with the *Copia* superfamily (Figure 3). *Copia-50_PHord* uses the anticodon loop of tRNA-Tyr as a primer, *Copia-18_PHord* uses that of tRNA-Ile, *Copia-6_PHord* uses that of tRNA-Asp, and *Copia-28_PHord* uses that of tRNA-iMet. *Copia-39_PHord* uses the 3′ end of tRNA-iMet as a primer for reverse transcription. This suggests that animal *Helenus* is mobilized by the *Gypsy* superfamily, while fungal *Helenus* is mobilized by the *Copia* superfamily of LTR retrotransposons. 

The priming of reverse transcription at the anticodon loop of tRNA is known for *Ty5* from *Saccharomyces cerevisiae* and *Copia* from *Drosophila melanogaster*, both belonging to the *Copia* superfamily of LTR retrotransposons [37,38]. A systematic analysis of plant LTR retrotransposons reported that the 3′-truncated tRNA primer is used only by several lineages in the *Copia* superfamily [39]. Finally, the lengths of TSDs of animal and fungal *Helenus* families show a clear distinction between them; animal *Helenus* families generate 4 bp TSDs, while fungal *Helenus* generates 5 bp TSDs. The *Copia* families showing similarity to *Helenus* PBSs generate 5 bp TSDs [3]. 

**Figure 4:**
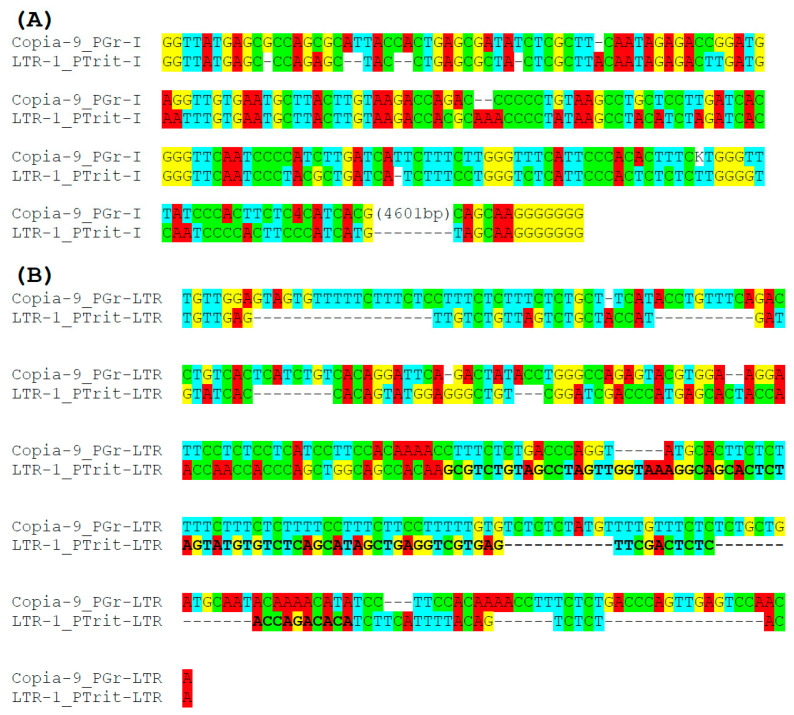
The alignment of *Copia-9_PGr* and *LTR-1_PTrit*. (**A**) Internal portion. (**B**) LTR. tRNA-like sequence is shown in bold.

The sequence similarity between *Copia-9_PGr* and *LTR-1_PTrit* is a more direct support for the mobilization of fungal *Helenus* by *Copia* (Figure 4). The 5′ 198 bp and the 3′ 12 bp sequences of the internal portion of *Copia-9_PGr* correspond to the entire internal portion of *LTR-1_PTrit*. Because *Copia-9_PGr* is from *P. graminis* and *LTR-1_PTrit* is from *P. triticina*, *LTR-1_PTrit* is not mobilized by *Copia-9_PGr*. More likely, a close relative of *Copia-9_PGr* transposes *LTR-1_PTrit*. *Helenus* families related to *LTR-1_PTrit* are distributed among the genera *Puccinia*, *Uromyces*, and *Cronartium*, indicating the coevolution between *Helenus* and *Copia*. LTRs of *Copia-9_PGr* and *LTR-1_PTrit* are not similar to each other. 

Different tRNA primer usage in fungal, hymenopteran, or molluscan *Helenus* lineages suggests the co-evolution between *Helenus* and its autonomous counterpart. As LTRs are not well conserved, a phylogenetic tree based on the tRNA-like sequences was produced for the fungal *Helenus* families (Figure 3). Due to the low conservation among *Helenus* families, the phylogeny does not have strong statistical support. The dominant tRNA primer type in the fungal *Helenus* families is the anticodon loop of tRNA-iMet-CAT. At least five lineages of *Helenus* changed their tRNA primer type. tRNA-Asn-GTT was chosen as a primer once in the evolution of fungal *Helenus* (Figure 3, green). tRNA-Ile-AAT was likely selected once (Figure 3, brown). The primer usage of tRNA-Tyr-GTA (Figure 3, blue) and the 3′ terminus of tRNA-iMet-CAT (Figure 3, purple) evolved multiple times. 

One might think that the tRNA-like sequence in the LTRs works as a primer for reverse transcription. However, we could not find any sequence complementarity between the tRNA-like sequence in the LTRs and its corresponding PBS. Thus, the tRNA-like sequence in the LTRs does not contribute to the reverse transcription initiation. 

### 3.7. Ajax: TRIM with a 5S rRNA Promoter

**Figure 5:**
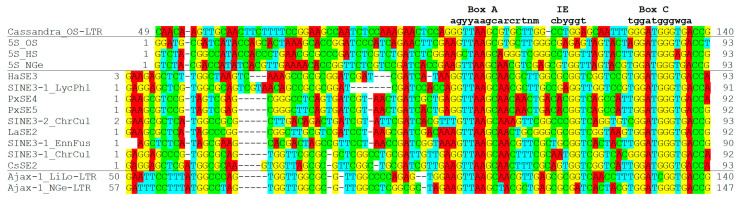
The sequence alignments of the 5S rRNA-like sequences in *Ajax*, *Cassandra*, and lepidopteran SINE3 along with 5S rRNA genes. Box A, IE and box C for RNA polymerase III promoter are shown above the alignment with their motif sequences. 5S rDNA sequences are from *Oryza sativa* (5S_OS), *Homo sapiens* (5S_HS), and *Notospermus geniculatus* (5S_NGe). Lepidopteran SINE3 are from *Helicoverpa armigera* (*HaSE3*), *Lycaena phlaeas* (*SINE3-1_LycPhl*), *Plutella xylostella* (*PxSE4* and *PxSE5*), *Chrysoteuchia culmella* (*SINE3-1_ChrCul* and *SINE3-2_ChrCul*), *Lerema accius* (*LaSE2*), *Ennomos fuscantarius* (*SINE3-1_EnnFus*), and *Chilo suppressalis* (*CsSE2*). The positions of the aligned sequence in the respective consensus is shown at both sides.

Routine screening of repetitive sequences from the nemertean *Notospermus geniculatus* revealed the presence of TRIMs whose LTR contains a fragment of the 5S rRNA gene (Figure 5). This TRIM lineage with a partial 5S rRNA-derived sequence in its LTRs is designated as *Ajax*. *Ajax* was found in two closely related nemerteans: *N. geniculatus* and *L. longissimus* (Table S2). The 5S rRNA-derived sequences in *Ajax* only cover the promoter region containing three motifs: box A, IE, and box C (Figure 3). As *Ajax* includes only a part of the 5S rRNA sequence, it is unlikely to encode a functional 5S rRNA. The secondary structure of LTR of *Ajax-1_LiLo* predicted by RNAfold (http://rna.tbi.univie.ac.at/) (last access: 28 November 2023) does not show similarity to the canonical structure of 5S rRNA. The sequence conservation of the promoter motifs suggests that the function of the 5S rRNA-derived sequence is to promote the transcription of *Ajax* by RNA polymerase III. 

It is speculated that *Cassandra* originated from the retrotransposition of a SINE3 element into an LTR, which was then copied into the other LTR by canonical retrotransposition [25]. *Ajax*, SINE3, and 5S rRNA sequences were compared to investigate the origin of the 5S rRNA-like sequence in *Ajax* LTRs (Figure 3). A Censor search against Repbase revealed the sequence similarity between *Ajax* and *HaSE3*, a SINE3 family from the lepidopteran insect *Helicoverpa armigera* [16]. It is unlikely that *HaSE3* contributed to the birth of *Ajax* considering the divergence of the host organisms, but the comparison could shed light on the evolution of *Ajax*. SINE3 families have been identified from other lepidopteran insects [40]. Four more SINE3 families were characterized here to compare the 5S rRNA-derived sequences; they are *SINE3-1_LyPhl* from the common copper brome *Lycaena phlaeas*, *SINE3-1_ChrCul* and *SINE3-2_ChrCul* from the garden grass-veneer *Chrysoteuchia culmella*, and *SINE3-1_EnnFus* from the dusky thorn *Ennomos fuscantarius*. The comparison revealed the higher sequence conservation in the promoter regions and the higher divergence in the upstream regions. It is noteworthy that *Ajax* shows a longer similar sequence to several SINE3 families: *CsSE2* and *SINE3-1_ChrCul*. This suggests that the 5S rRNA-like sequence in *Ajax* is not a direct descendant of 5S rRNA. The sequence upstream of the promoter regions of *Ajax* might have diverged in the ancestral SINE3 family, then integrated into an LTR of the ancestor of *Ajax*. 

The sequence downstream of the 5′ LTR is complimentary to the 3′ end of tRNA-Leu (Figure S3), indicating that tRNA-Leu works as a primer for reverse transcription. 

### 3.8. Copy Number Estimates of Helenus and Ajax

The copy numbers of *Helenus* and *Ajax* were estimated from the six genomes of divergent species: *P. triticina*, *C. ribicola*, *D. rerio*, *C. gigas*, *N. vitripennis*, and *L. longissimus* (Table S3). Here, the sequences longer than 90% of the respective consensus were examined. The copy number of LTRs could be higher than twice that of internal portions, considering the presence of solo LTRs. The largest *Helenus* family in the two species of fungi is *LTR-1_PTrit*, which contains 529 LTRs and 144 internal portions. Small families such as *Helenus-10_PTrit* and *Helenus-12_PTrit* retain less than 10 LTRs. In the four analyzed animal species, *LTR-11_DR* shows enormous copies: 5575 LTRs and 2618 internal portions. Tandem arrays of *LTR-11_DR* are observed. *Helenus-2C_CGi* retains 1124 LTRs and 402 internal portions. *Ajax-1_LiLo* has 1114 LTRs and 151 internal portions.

## 4. Discussion

### 4.1. TRIMp3—Proposed TRIM Families Containing an RNA Polymerase III Promoter

*Helenus*, *Ajax*, and *Cassandra* [25] represent a unique type of TRIMs; they include a small RNA gene-derived sequence in their LTRs. *Cassandra* produces transcripts from the RNA polymerase III promoter. Without experimental validation, it cannot be concluded that *Helenus* and *Ajax* contain a functional RNA polymerase III promoter, but the sequence conservation indicates the functionality. *Ajax* includes only a part of the 5S rRNA sequence, likely the part only responsible for promoting the transcription by RNA polymerase III. *Helenus* is unlikely to encode a functional tRNA either. These observations indicate that although they do not exclude the possibility that these TRIM families encode a functional RNA, the main selective drive for such TRIMs is propagating themselves, not contributing to the repertoire of cellular RNA components. 

These TRIMs share some properties with SINEs. Both contain a small RNA-derived sequence, likely functioning as an RNA polymerase III promoter. Because retrotransposon RNA works as a template for reverse transcription as well as for protein synthesis, the RNA length is a restriction factor for the retrotransposon length. The length of SINEs is restricted, related to the weak processivity of RNA polymerase III compared with RNA polymerase II. SINEs are 100 to 700 bp [11]. *Helenus* is between 311 and 1263 bp in length. *Ajax* is 421 (*Ajax-1_LiLo*) or 434 bp (*Ajax-1_NGe*). *Cassandra* is from 565 to 860 bp in length [25]. Thus, the length of these TRIMs seems also restricted by the processivity of RNA polymerase III. 

Here, it is proposed to call the TRIM families containing an RNA polymerase III promoter together “TRIMp3.” *Cassandra* is the only validated TRIMp3 lineage to date, and *Helenus* and *Ajax* are expected to be added. Canonical TRIM families can be called “TRIMp2.” We can speculate a TRIM family with a 7SL RNA sequence. SINE1 has been found only in primates, rodents, and hagfishes [13,15,41]. It seems related to the reasons why a TRIM family with a 7SL RNA-derived sequence has not been found yet. 

SINEs work as an enhancer, promoter, insulator, splicing signal, and transcription factor-binding site [42,43]. As TRIMp3 and SINEs are both short interspersed repetitive elements that remain a promoter recruiting RNA polymerase III, it is likely that many biological functions of SINEs can also be applied to TRIMp3. Rapid transcriptional regulatory rewriting mediated by TRIMp3 mobilization might happen in TRIMp3-rich lineages such as Pucciniales fungi and parasitoid wasps. 

### 4.2. The Evolution of TRIMp3

SINEs are mobilized by non-LTR retrotransposons, while TRIMs are mobilized by LTR retrotransposons. The presence of the PBS and PPT in *Helenus* and *Ajax* supports that their retrotransposition mechanism is identical to that of most LTR retrotransposons. A tRNA primer works for the first-strand DNA synthesis, and the PPT contributes to the second-strand synthesis. 

It is almost certain that *Ajax* and *Cassandra* have independent origins. The wide distribution of *Cassandra* in green plants suggests that they originated in the common ancestor of angiosperms and ferns, over 250 MYA [25]. *Ajax* is found only in two nemertean species and likely originated in this lineage. The divergent sequence upstream of the promoter motifs in *Ajax* LTRs (Figure 5) suggests that they originated by SINE3 insertion into the ancestral TRIM, as proposed for *Cassandra*. 

SINE2 is much more abundant than SINE3, so it is also likely that *Helenus* was born multiple times through SINE2 insertion into an LTR of the ancestral TRIM. Their patchy distribution and diversity also support their multiple origins. Several lines of data suggest that fungal *Helenus* families depend on the *Copia* superfamily of LTR retrotransposon for their mobilization, while animal *Helenus* depend on the *Gypsy* superfamily of LTR retrotransposon. It is speculated that molluscan *Helenus* in the subclass Pteriomorphia originated > 470 MYA, fish *Helenus* in the superorder Ostariophysi ~200 MYA, fungal *Helenus* in the order Pucciniales ~130 MYA, and hymenopteran *Helenus* in the superfamily Chalcidoidea > 100 MYA in their respective ancestors. 

During the long history of the evolution of *Helenus*, there have been many diversification events. Multiple *Helenus* families can co-exist in a single genome (Table S1). The speciation of *Helenus* families can occur independently from their counterpart LTR retrotransposons. In that case, multiple *Helenus* families depend on a single family of LTR retrotransposon for mobilization. It is also possible that the speciation of *Helenus* occurs along with the diversification of its counterpart LTR retrotransposon. Such co-speciation events could have happened given the observed PBS diversity. Although the detailed mechanism is unknown, LTR retrotransposons have changed their tRNA primer type during evolution [39]. Fungal *Helenus* also shows the change in tRNA primer type during evolution (Figure 3). Other groups of *Helenus* also show the diversity of PBS, so they also have experienced the change in tRNA primer type. The change in tRNA primer type by *Helenus* could have happened simultaneously with the change by its counterpart LTR retrotransposon. Autonomous *Copia* families reported from the genome of *P. hordei* show all varieties of tRNA primers used by fungal *Helenus* families (Figure 3). Long-term maintenance of *Helenus* must coincide with the continuous presence of active autonomous counterparts. Co-evolution between *Helenus* and its autonomous counterpart is the most likely scenario to explain their maintenance and diversity. Or *Helenus* could have shifted its tRNA primer type by changing its autonomous counterpart. The change in autonomous counterpart is common in SINE evolution [15]. 

It is not known how the dependence of TRIM on its autonomous LTR retrotransposon is maintained. The sequence similarity in LTRs between TRIMs and autonomous LTR retrotransposons is an indication of their relationships [19]. Some of these pairs also show sequence similarity in the internal portions between two LTRs. In the case of SMARTs, while there is no sequence similarity between the LTRs of SMARTs and their putative autonomous counterpart FRetro64, the internal portion of SMARTs shows high sequence identity to a part of FRetro64 [18]. Considering the weak processivity of RNA polymerase III, their counterpart autonomous LTR retrotransposons must use a promoter for RNA polymerase II similar to canonical LTR retrotransposons. Therefore, it is unlikely that a long stretch of sequence is shared between TRIMp3 and its autonomous counterpart in their LTRs. In fact, *LTR-1_PTrit* and related *Helenus* families include a sequence similar to the internal portion but not the LTRs of *Copia-9_PGr* (Figure 4). Although *Cassandra* also has a long history of evolution and likely has diversified into multiple lineages, we have no indication for the autonomous LTR retrotransposon family responsible for *Cassandra* mobilization. Another unique group of non-autonomous LTR retrotransposons, called retrozymes, also contain LTRs that do not share sequences with canonical LTR retrotransposons [44]. These observations suggest that the homology of LTRs is not necessary for trans-mobilization. Many recently active *Copia* families have been reported from various *Puccinia* species and *Gypsy* families from *C. gigas* [3]. They could be a source for further studies of the evolution, diversification, and mobilization of TRIMp3 families.

## 5. Conclusions

Here, two new groups of non-autonomous LTR retrotransposons are characterized: *Helenus* and *Ajax*. *Helenus* is found in fungi, animals, and plants and contains a tRNA-like sequence in its LTRs. *Ajax* is found in nemertean species and includes a short sequence corresponding to the promoter of the 5S rRNA gene. The finding of *Helenus* and *Ajax* suggests the presence of short non-autonomous LTR retrotransposons (TRIMs) with a small RNA-derived sequence functioning as a promoter for RNA polymerase III, here designated TRIMp3. These small RNA-derived sequences in TRIMp3 likely function to produce a template RNA for reverse transcription. There may be a direct relationship between TRIMp3s and SINEs; a SINE insertion into an LTR of TRIM may lead to the birth of TRIMp3. 

## Figures and Tables

**Table 1:** The distribution of *Helenus*.

Kingdom	Phylum	Class	Order	Family	Species
Fungi	Basidiomycota	Pucciniomycetes	Pucciniales	Pucciniaceae	*Puccinia graminis*, *P. striiformis*, *P. triticina*, *P. hordei*, *P. horiana*, *P. coronata*, *P. novopanici*, *P. sorghi*, *P. arachidis*, *P. polysora*, *Uromyces transversalis*, and *U. viciae-fabae*
				Sphaerophragmiaceae	*Austropuccinia psidii*
				Melampsoraceae	*Melampsora larici-populina*, *M. aecidioides*, and *M. medusae*
				Coleosporiaceae	*Cronartium ribicola*
				Zaghouaniaceae	*Hemileia vastatrix*
		Agaricomycetes	Agaricales	Agaricaceae	*Agaricus bisporus*
				Psathyrellaceae	*Coprinopsis cinerea*
Metazoa	Ctenophora	Tentaculata	Lobata	Bolinopsidae	*Mnemiopsis leidyi*
	Chordata	-	Coelacanthiformes	Coelacanthidae	*Latimeria chalumnae*
		Actinopteri	Acipenseriformes	Acipenseridae	*Acipenser ruthenus*
				Polyodontidae	*Polyodon spathula*
			Cypriniformes	Danionidae	*Danio rerio*
				Cyprinidae	*Cyprinus carpio*, *Carassius auratus*, *Culter alburnus*, *Onychostoma macrolepis*, *Phoxinus phoxinus*, and *Squalius cephalus*
				Nemacheilidae	*Triplophysa dalaica*
			Siluriformes	Bagridae	*Hemibagrus wyckioides*
			Gonorynchiformes	Chanidae	*Chanos chanos*
	Mollusca	Bivalvia	Ostreida	Ostreidae	*Crassostrea gigas*, *C. virginica*, and *Saccostrea glomerata*
			Pterioida	Pinnidae	*Pinna nobilis*
				Pteriidae	*Pinctada imbricata*
			Mytilida	Mytilidae	*Mytilus galloprovincialis*, *M. coruscus*, *Modiolus philippinarum*, *Gigantidas (Bathymodiolus) platifrons*, and *Limnoperna fortunei*
			Pectinida	Pectinidae	*Argopecten irradians*, *Mizuhopecten yessoensis*, and *Pecten maximus*
			Arcoida	Arcidae	*Tegillarca granosa*
	Brachiopoda	Lingulata	Lingulida	Lingulidae	*Lingula anatina*
	Nemertea	Pilidiophora	Heteronemertea	Lineidae	*Lineus longissimus* and *Notospermus geniculatus*
	Arthropoda	Insecta	Hymenoptera	Pteromalidae	*Nasonia vitripennis*, *Cecidostiba fungosa*, *Trichomalopsis sarcophagae*, *Muscidifurax raptorellus*, *Philotrypesis tridentata*, *Theocolax elegans*, *Gastracanthus pulcherrimus*, and *Pteromalus puparum*
				Eurytomidae	*Eurytoma adleriae*
				Eupelmidae	*Eupelmus annulatus*
				Megastigmidae	*Megastigmus dorsalis*
				Cynipidae	*Synergus japonicus* and *S. umbraculus*
				Torymidae	*Torymus geranii*
Viridiplantae	Streptophyta	Lycopodiopsida	Selaginellales	Selaginellaceae	*Selaginella moellendorffii*

**Table 2:** *Helenus* and tRNA genes predicted in GtRNADB.

Species	tRNA Type	Total	*Helenus*	Not *Helenus*
*Puccinia triticina*	tRNA-Thr-AGT	492	481	11
			(*LTR-1_PTrit*: 426)	
			(*Helenus-8_PTrit*: 55)	
	tRNA-Ser-TGA	189	183	6
			(*Helenus-2_PTrit*: 95)	
			(*Helenus-11_PTrit*: 40)	
			(*Helenus-11B_PTrit*: 33)	
			(*Helenus-9_PTrit*: 11)	
	tRNA-Ala-TGC	137	133	4
			(*Helenus-4_PTrit*: 133)	
	tRNA-Pro-TGG	128	123	5
			(*Helenus-5_PTrit*: 123)	
*Puccinia striiformis*	tRNA-Ala-AGC	163	147	16
			(*LTR-2_PSt*: 145)	
	tRNA-Thr-AGT	49	32	17
			(*Helenus-4_PSt*: 21)	
	tRNA-Ser-AGA	44	28	16
			(*Helenus-9_PSt*: 28)	
*Puccinia graminis*	tRNA-Thr-AGT	100	89	11
			(*Helenus-3_PGr*: 58)	
			(*Helenus-4_PGr*: 15)	
*Uromyces viciae-fabae*	tRNA-Thr-AGT	119	112	7
			(*Helenus-1_UVF*: 94)	
	tRNA-Sup-CTA	109	109	0
			(*Helenus-1_UVF*: 105)	
	tRNA-Ser-AGA	95	90	5
			(*Helenus-2_UVF*: 88)	
*Cronartium ribicola*	tRNA-Ala-AGC	258	251	7
			(*Helenus-4_CroRib*: 249)	
	tRNA-Sup-CTA	245	245	0
			(*Helenus-5_CroRib*: 194)	
			(*Helenus-3_CroRib*: 51)	
	tRNA-Thr-AGT	160	154	6
			(*Helenus-2_CroRib*: 88)	
			(*Helenus-4_CroRib*: 29)	
			(*Helenus-5_CroRib*: 22)	
	tRNA-Ser-AGA	160	154	6
			(*Helenus-6_CroRib*: 118)	
			(*Helenus-13_CroRib*: 13)	
	tRNA-Val-GAC	127	126	1
			(*Helenus-16_CroRib*: 126)	

## Data Availability

The data presented in this study are available in the Supplementary Materials.

## Supplementary Materials

The following supporting information can be downloaded at: https://www.mdpi.com/article/10.3390/biology13020119/s1, Figure S1: Structural alignment of predicted tRNA sequences encoded in *Helenus*; Figure S2: Primer-binding sites (PBSs) of *Helenus* families; Figure S3: Primer-binding sites (PBSs) of *Ajax* families; Table S1: List of *Helenus* families; Table S2: List of *Ajax* families; Table S3: Copy numbers of *Helenus* and *Ajax* families from six representative organisms. Data S1: Consensus sequences of *Helenus* and *Ajax* families reported in this study in the fasta format.
