# Supplementary material for: Helenus and Ajax, Two Groups of Non-Autonomous LTR Retrotransposons, Represent a New Type of Small RNA Gene-Derived Mobile Elements
Source: Biology (Basel). 2024 Feb 13;13(2):119. doi: 10.3390/biology13020119 (PMC10886601; doi:10.3390/biology13020119)

Figure S1. Structural alignment of predicted tRNA sequences encoded in *Helenus*. Anticodons are in bold. Possible introns are in lowercase. Predicted secondary structure is shown below each tRNA-like sequence. Numbers indicate the positions inside of LTR.

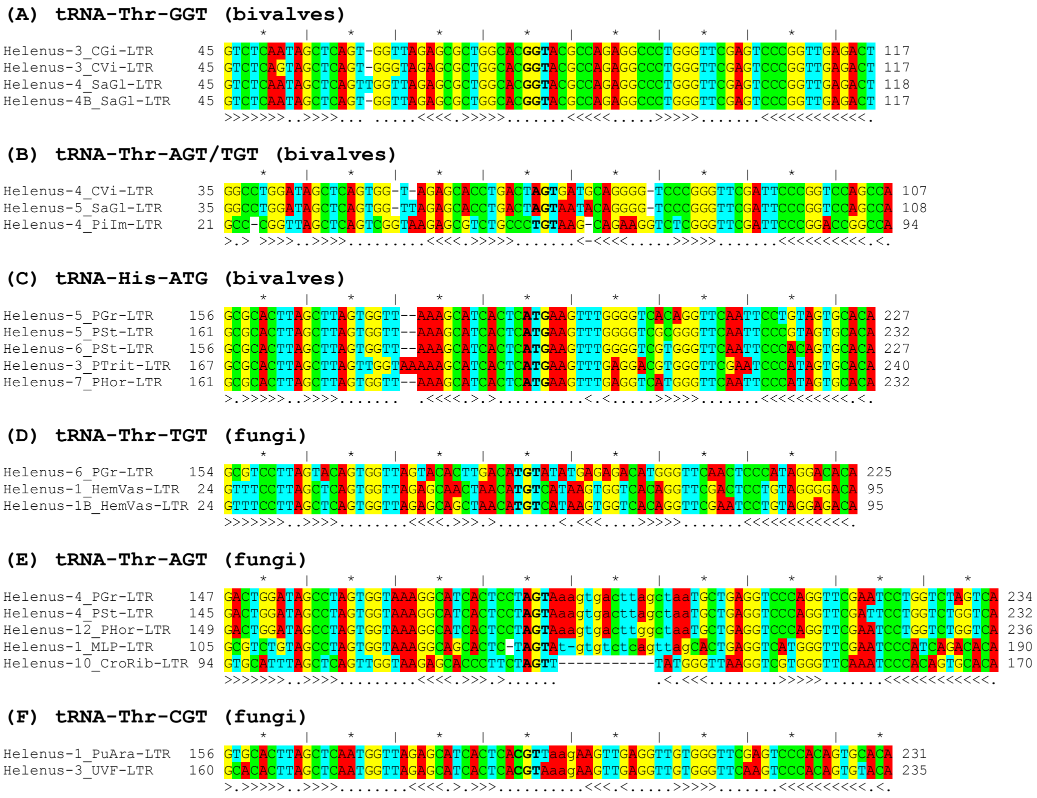

Supplement: Supplementary file 1 [file biology-13-00119-s001.zip › FigS1-Helenus-tRNA-funcB.pdf]
