# Supplementary material for: Helenus and Ajax, Two Groups of Non-Autonomous LTR Retrotransposons, Represent a New Type of Small RNA Gene-Derived Mobile Elements
Source: Biology (Basel). 2024 Feb 13;13(2):119. doi: 10.3390/biology13020119 (PMC10886601; doi:10.3390/biology13020119)
Supplement: Supplementary file 1 [file biology-13-00119-s001.zip › FigS2_HelenusPBS.pdf]

Figure S2. Primer-binding sites (PBSs) of *Helenus* families. The tRNA sequence is shown in red in the reverse orientation. The enzymatically added 3’ terminal CCA is shown in lowercases. The PBS nucleotides complementary to the tRNA are highlighted in yellow. **(A)** *Helenus* from hymenopteran insects. **(B)** *Helenus* from vertebrates. **(C)** *Helenus* from bivalves. tRNA sequences are from *Apis mellifera* for hymenopteran insects, *Homo sapiens* for vertebrates, and *Aplysia californica* for bivalves.

(A)

```
tRNA-Lys-CTT        3’-accGCGGGUUGCACCCCGAG-5’ (A. mellifera)
BEL-4_NVi-I.        TTTTGGCGCCCGAACAGGGACCT
Gypsy-16-I_NVi        TTGGCGCCCAGTTCGTGGGTT
Helenus-1_MeDo-I       TGGCGCCCAACTAAACGACC
Helenus-1_PhiTri-I     TGGCGCCCAACTAATAAATC
Helenus-2_EuAn-I     ACTGGCGCCCAACTAAATAACC
Helenus-4_MusRap-I     TGGCGCCCAACTGAAATACC
Helenus-1_MusRap-I     TGGCGCCCAACTGAATAACC
ULTR-1-I_NVi           TGGCGCCCAACTGAATTACC
Helenus-2_PhiTri-I   AATGGCGCCCAACTCAATAACC
Helenus-2_NVit-I       TGGCGCCCAACTCAAAAACC
Helenus-3_PtePup-I     TGGCGCCCAACTCAAAAACC
Helenus-3_TheEle-I   AATGGCGCCCAACTCAAAAACC
Helenus-2_EuAd-I      TTGGCGCCCAACTAAAAAACC
Helenus-1_EuAd-I      TTGGCGCTCAACTAAAAAACC
Helenus-2_PtePup-I     TGGCGCTCAACTGAATTACC
Helenus-1_TrSa-I       TGGC-CCCAACTGAATTACC

tRNA-Leu-AAG        3’-accACCGUCGCCACCCU-5’ (A. mellifera)
Gypsy-130_CGi-I  TAAATTTGGTGGCAGCGAGCGGT
Gypsy-57_CaAu-I       TTGGTGGCAGCGGTGGGA
Helenus-2_TheEle-I  AAGTGGTGGCAGCTAATGGG

tRNA-Leu-CAG        3’-accACAGUCUUCACCCU-5’ (A. mellifera)
Gypsy-28-I_NVi      TTTTGGTGTCAGAATTGGTT
Helenus-3_EuAn-I    TTCTGGTGTCAGAAGCAGGA

tRNA-Trp-CCA        3’-accACUGGGGCUGCACUAA-5’ (A. mellifera)
Gypsy-32-I_NVi        TTGGTGACCCCGACGTGATT
Helenus-2_SyJa-I      TTGGTGACCCCGACACTGCT

tRNA-Arg-TCT        3’-accGUAGGGAACGCCCCUA-5’ (A. mellifera)
Gypsy-31_NVi-I       TCTGGCATCCCTACGTGGGG
Helenus-1_TheEle-I   GATGGCATCCCTTGTGTGTC
Helenus-2_MusRap-I  TTCTGGCATCCCTTGTGGGGA
Helenus-2_GasPul-I  AAGTGGCATCTCTTGTGGGGA
Helenus-1_GasPul-I  TTCTGGCATMCCTTGTGCAGG
Helenus-4_NVit-I     CTTGGCATCCCTTTGTGCGG
Helenus-1_PtePup-I   CTTGGCATCCCTTCGTGCGG
Helenus-3_NVit-I    TTCTGGCATCCCTTACGGGGG
Helenus-1_CeFu-I     CTTGGCATCCCTGATGGGGA
Helenus-2_TrSa-I    TTCTGGCATCCCTGGTGCGGA
Helenus-3_PhiTri-I  TTCTGGCATCCCTGGTACGGG
```

(B)

```
tRNA-His-GTG        3’-accACGGCACUGAGCCUAA-5’ (H. sapiens)
Helenus-1_TrDa-I      TTTGTGCCGTGACCCGGATT
Helenus-1_ChCh-I  TACATTGGTGCCGTGACCCGGATG
LTR-11_DR-I           TTGGTGCCGTGACCCGGATG

tRNA-Leu-CAA        3’-accACAGUCUUCACCCUAAGCUU-5’ (H. sapiens)
Helenus-1_AcRu-I      TTGGTGTCAGAAGTGGGATG
Helenus-1_PolSpa-I    TTGGTGTSAGAAGTGGGACG

tRNA-Lys-CTT        3’-accGCGGGUUGCAUCCCGAGUUU-5’ (H. sapiens)
LTR1_LCh              ATGGCGCCCAACTCGATGGCTT
LTR2_Lch              ATGGCGCCCAACTCGATGGCTT
```

(C)

```
tRNA-Lys-CTT               3’-accGCGGGUUGCUCCCCG-5’  (A. californica)
Gypsy-125_CGi-I             AATGGCGCCCAACGTGATTG
Helenus-3_CGi-I             TTTGGCGCCCAACGTACAAA
Helenus-4_SaGl-I            TTTGGCGCCCAACGTACAAA
Helenus-3_CVi-I             TTTGGCGCCCAACGTAGATA
Helenus-1_PiIm-I             ATGGCGCCCAACGTAAATC
Helenus-4B_SaGl-I           TTTGGCGCCCAACGAAAATA
Helenus-1_PiNo-I            AATGGCGCCCA-CGTTGGGA
Helenus-1_MiYe-I             TTGGCGCCCA-CGTAGGGA
Helenus-3_MiYe-I             TTGGCGCCCA-CGTAGGGA
Helenus-1_CGi-I             TTTGGCGCCCA-CGTTGGGA
Helenus-3_SaGl-I            TTTGGCGCCCA-CGTTGGGA
Helenus-2_ArIr-I             TTGGCGCCCA-TGTAGGGA
Helenus-1_PeMa-I             TTGGCGCCCA-TGTAGGGA
Helenus-2_CVi-I             TTTGGCGCCCA-TGTTGGGA
Helenus-1_LimFor-I          TTTGGCGCCCAACTGAAAAA
Helenus-3_MyGa-I            TTTGGCGCCCAACTAAAAAC
Helenus-6_SaGl-I             TTGGCGCCCAACTAAAAAA
Helenus-1_MyCo-I             TTGGCGCCCAACTAAAAAA
Helenus-2_PeMa-I         AAATTGGCGCCCAACTAAAATA
Helenus-5_PiIm-I          GAATTGGCGCCAAACTAAATGA
Helenus-3_ArIr-I             TTGGCGCCCAACTAAATAA
Helenus-1_BaPl-I             TTGGCGCCCAACTAAATAA
Helenus-1_ArIr-I         GAATTGGCGCCCAACTAAATAA
Helenus-1_MyGa-I             TTGGCGCCCAACAATAATA
Helenus-1_MoPh-I            TTTGGCGCCCAGCAAAAAAC
Helenus-1B_MoPh-I           TTTGGCGCCCAGCAAAAAAC

tRNA-His-GTG               3’-accACGGCA-CUGGUCCUAAGC-5’  (A. californica)
Gypsy-39_CGi-I.          TTATTTGGTGCCGT-GACCAGGA
Helenus-3_PeMa-I             TTGGTGCCGCAGACCACTC
Helenus-4_ArIr-I            TTTGGTGCCGTAGACCACTC
Helenus-2_SaGl-I    ------GATTTGGTGCCGTTGACCACCC
Helenus-4_PeMa-I    ------GATTTGGTGCCGTTGACCACTC
Helenus-5_PeMa-I            TTTGGTGCCGTTGACCACTC
Helenus-2C_CGi-I            TTTGGTGCCGT-GACCAACC
Helenus-1_SaGl-I            TTTGGTGCCGT-G-CCATGC
Helenus-4_MiYe-I    -------AATTGGTGCCGTTGACCCTC-
Helenus-2_CGi-I             TTTGGTGCCGT-GACCTGCC
Helenus-1_CVi-I             TTTGGTGCCGT-GACCTGCC
Helenus-2B_CGi-I            GATGGTGCCGT-GGCCTTCC
Helenus-2_PiIm-I            TTTGGTGCCGT-GAGAGTGA

tRNA-His-GTC               3’-accAC-GGCACUGGUCCUA-5’  (A. californica)
Gypsy-39_CGi-I           TTATTTGGTG-CCGTGACCAGGAT
Helenus-4_PiIm-I    GTACAGTATTTGGTG-CCGTGAGAGTGAA
Helenus-4_CVi-I     --------TTTGGTG-CCATGGCCTTCCC
Helenus-2_MiYe-I    ----------TGGTGTCCGTGGCCCTCCC

tRNA-Ile-AAT               3’-accACCGGUCAUACCCCC-5’  (A. californica)
Gypsy-167_CGi-I            TTTGGTGGCCAGTGTGGGGG
Helenus-5_SaGl-I    --------TTTGGTGGCCAGTGTGGGGG
Helenus-2_TeGr-I    -------GTTTGGTGGCCAGTGTGGCCC

tRNA-Pro-AGG               3’-accCCCGAGCAGGCCCUA-5’  (A. californica)
Gypsy-13_CGi-I      --TAAATATTTGGGGGCTCGCTCCGAGG
Helenus-1_TeGr-I    --------TTTGGGGGCTCGTCCAACCC

tRNA-Glu-NNN               3’-accGCAGCGAUGGGCCUAA-5’  (A. californica)
Helenus-2_PiNo-I    ---------CTGGCGTCGCCGGTAAATGG
Helenus-3_PiNo-I    ---------ATGGCGTCGCCGGAAAACCG

tRNA-Ser-TGA               3’-accGCGACGGCCGUCCUAA-5’  (A. californica)
Helenus-2_LimFor-I  ---------TTGGCGCTGCGAGCAACAAC
Helenus-2_MyGa-I    ---------TTGGCGCTGCGAGCAATTAC
Helenus-2_MoPh-I    ---------TTGGCGCTGCGAGCACTTAC

tRNA-Ser-GCT               3’-accGCUGCUCCUACCCUAA-5’  (A. californica)
Helenus-3_PiIm-I    ---------TTGGCGACGAGGTGAACAAT
Helenus-6_PiIm-I    ---------CTGGCGACGAGGTGAACAAT
```
