# Supplementary material for: Helenus and Ajax, Two Groups of Non-Autonomous LTR Retrotransposons, Represent a New Type of Small RNA Gene-Derived Mobile Elements
Source: Biology (Basel). 2024 Feb 13;13(2):119. doi: 10.3390/biology13020119 (PMC10886601; doi:10.3390/biology13020119)
Supplement: Supplementary file 1 [file biology-13-00119-s001.zip › FigS3_AjaxPBS.pdf]

Figure S3. Primer-binding sites (PBSs) of *Ajax* families. The tRNA sequence is shown in red in the reverse orientation. The PBS nucleotides complementary to the tRNA are highlighted in yellow. tRNA sequences are from *Homo sapiens*.

```
tRNA-Leu-AAG           3’-ACCGUCGCCACCCUAAG-5’ (H. sapiens)
Ajax-1_NGe-I              TCTGGCAGACCTTCCCAGTC
Ajax-1_LiLo-I             TCTGGCAGACCTTCCCACGC
```
